# Correction: New measures of agency from an adaptive sensorimotor task

**DOI:** 10.1371/journal.pone.0247933

**Published:** 2021-02-25

**Authors:** The PLOS ONE Staff

There is an error in the article title. The correct title is: Measuring agency with an adaptive sensorimotor task. The publisher apologizes for the error.

The correct citation is: Wang S, Rajananda S, Lau H, Knotts JD (2020) Measuring agency with an adaptive sensorimotor task. PLoS ONE 15(12): e0244113. https://doi.org/10.1371/journal.pone.0244113
