# Supplementary material for: New measures of agency from an adaptive sensorimotor task
Source: PLoS One. 2020 Dec 21;15(12):e0244113. doi: 10.1371/journal.pone.0244113 (PMC7751868; doi:10.1371/journal.pone.0244113)
Supplement: Attachment — Submitted filename: Response to Reviewers.docx [file pone.0244113.s002.docx]

**Response to Reviewers for PONE-D-20-24359: New measures of agency from an adaptive sensorimotor task**

We thank the editor and reviewers for considering our manuscript for publication, and for their valuable comments. We have edited the manuscript to address the comments below, particularly the concerns about the effectiveness of the task, providing more details about the task, justifying the calculation of test-retest reliability, and reporting analyses on cursor movements. Below, the reviewer comments are shown in blue, our responses are in black, and excerpts of the revised manuscript are in italics, with changes shown in red. We thank the editor and reviewers again for their time.

## Reviewer #1

1. It is very concerning that almost half the participants in each experiment had to be excluded. A test that yields usable data only half the time does not seem like it would have much clinical utility.
   a. Why would so many people miss more than 40% of the catch trials?

We thank the reviewer for this comment. We agree that this high exclusion rate could lead to concerns about the effectiveness of the task.

To address this concern, we performed multiple independent t-tests to compare the control thresholds of participants who missed more than 40% of the catch trials with those who missed less than 40% of the catch trials. The results showed that subjects who were excluded due to low catch trial accuracy had significantly higher control thresholds than subjects who were not excluded by the catch trial criteria, in both experiment 1 and 2 (see statistics below). Moreover, for both experiments, one-sample t-tests indicated that the control thresholds of excluded participants were significantly lower than the control level of catch trials (25%).

These results implied that the excluded participants have the ability to perform well on the catch trials but failed to do so potentially due to their lack of attention to the task. These observations are consistent with previous evidence that mTurk participants exhibit lower levels of attentiveness compared to in-person participants (Goodman et al., 2012).

Although the observations above indicate that participant engagement may be a concern for the task’s efficacy, arguably, this should not be a concern when giving the task to patients as part of a diagnostic assessment. While healthy subjects performing the task are mainly incentivized by monetary reward (which were relatively low in our study -- up to $5), patients may have higher motivation and pay more attention to the task as it is closely related to their health. Thus, we expect the exclusion rate to be lower in clinical populations.

In a future study, we will give the current task to naive healthy subjects and patients with functional movement disorders (FMD) and compare the

performance of the two populations. The effectiveness of the task in both populations is expected to be better addressed by this future study.

The following revisions to the manuscript were made to reflect the response

above:

In the result section:

# *Previous studies suggest that, compared to in-person subjects, online participants* are less attentive to tasks, potentially resulting in poor data quality [36, but see also 37]. To control for this, we excluded participants based on their attentiveness, which was measured by their accuracy on the catch trials.

*Participants who missed more than 40% of the catch trials (Experiment 1: M = 7.67%, SD = 2.07%; Experiment 2: M = 6.97%, SD = 2.40%) had significantly higher control thresholds than participants who meet the catch trial criteria (Experiment 1: M = 2.55%, SD = 3.09%, t(94.332) = 9.77, p < .0001; Experiment*

*2: M = 2.21%, SD = 2.62%, t(55.469) = 8.57, p < .0001). Meanwhile, the*

*thresholds of participants who were excluded by the catch trial criteria were significantly lower than the control level of catch trials (25%) in both experiments (Experiment 1: t(36) = -51.03, p < .0001; Experiment 2: t(27) = -39.80, p <*

*.0001). These analyses imply that the excluded subjects were capable of performing well on the catch trials but failed to do so potentially due to their lack of attention to the task.*

In the discussion section:

# *Lastly, the high exclusion rate in the present study may raise concerns about the* effectiveness of the task; a task that yields usable data only 34.72% of the time would not seem to have much clinical utility. However, this concern is likely

*unfounded for a few reasons. First, high exclusion rates due to a lack of attentiveness to the task among mTurk participant samples have been reported previously [48, 49, but see also 37]. Indeed, the significantly higher control thresholds observed among excluded participants putatively indicates a lack of engagement among this group. Thus, administering the task to non-mTurk participants, such as college students, may lower exclusion rates. Moreover, common methods of increasing participants’ motivation, such as providing higher monetary rewards or gamifying the task could also be applied in future experiments. Importantly, we also expect that clinical patients will be less likely to have issues with reduced motivation or engagement, as the task is directly related to their condition.*

b. How long does the test take, on average? I don’t think that was mentioned in the manuscript. Is it long enough that many participants get bored?

This is a great question, and we agree that the length of the task should be reported in the manuscript.

Most of the participants finished the task in roughly 20 minutes (see the descriptive statistics in the revised section of the manuscript below). It is possible that participants may get bored when performing the task since each trial has the same structure.The low level of motivation and attentiveness of online participants may further accelerate this process. However, we believe that the reduction of participants’ interest in the task over time should not be a concern when the participants are from the clinical population (see response to comment #1a).

To address the comment, following changes were made to the manuscript:

# *On average, participants finished the task in about 20 minutes (Experiment 1: M*

*= 17.36 mins, SD = 9.34 mins; Experiment 2: M = 22.94, SD = 10.93).*

c. Do the authors have plans to make the test more engaging or other changes that might reduce the number of exclusions? These issues should at least be discussed.

We thank the reviewer for this suggestion and agree that potential solutions to the high exclusion rate should be discussed. Thus, in the revised manuscript, we discuss solutions such as increasing monetary incentive and gamifying the task. Our collaborators at University of Florida (led by Dr. Brian Odegaard [[bodegaard@ufl.edu](mailto:bodegaard@ufl.edu)]) ran this dot task with college students not on mTurk. He observed an exclusion rate of 9.52% (N = 21), which is lower than ours (Experiment 1: 39.39%; Experiment 2: 29.79%). Thus, we also recommended collecting data from non-mTurk users, such as college students, as one method of reducing the exclusion rate. However, we would like to again emphasize that clinical populations may have lower exclusion rates due to an expected increase in motivation given the task’s putative clinical relevance. Please see our response to comment 1a for the relevant manuscript revisions.

2. More details about the task are needed.
   a. Did the two dots perform identical movements, apart from the cursor movement? Or did each dot have its own random motion?

We agree with the reviewer that more details about the task should be provided. Apart from the cursor movement, the two dots perform random movements with independent trajectories, which are generated following the description in the method section.

To clarify this point, the following revision was made in the methods section of the manuscript:

# *On each trial, two moving dots (dot A and dot B) were presented within separate* circles for 4 or 2.5 seconds, in Experiment 1 and Experiment 2, respectively (Fig 1A, Video S1). Each dot had an independent, pseudorandom trajectory, meaning that without the influence of cursor movements, the two dots will have different random trajectories.

b. How were participants instructed to move the cursor?

We thank the reviewer for this comment. At the beginning of the task, participants were told that their cursor movement can influence one of the two dots on each trial, and their mission is to identify the dot that could be influenced. No specific

instructions on how the cursor should be moved were provided. We did this to avoid biasing participants towards specific strategies that could have potentially hindered their ability to identify the target dot.

To address this comment, we have revised the manuscript to include the exact instructions provided to the participants and to discuss the rationale above:

# *While the dots were presented, the participant could move the cursor to influence* the trajectory of one of the two dots, hereafter referred to as the target dot. The instruction participants received regarding cursor movements stated: “On each trial, you will see two independent moving dots. The movement of your mouse can influence the trajectory of one of them … Your goal is to report which dot you are better able to control and how confident you are on your decision.” No specific strategy for mouse movement was provided to avoid potentially biasing participants (e.g., toward shaking the cursor rapidly from side to side). The target dot (A or B) was determined randomly at the beginning of each trial.

c. What if they don’t bother moving the cursor and just guess the answers? Were such trials excluded?

We thank the reviewer for raising this point and agree that intermixing trials where participants actively participated with those in which they did not could affect the accuracy of self-agency measurements.

We did not record cursor movements in the current experiments, which limited our ability to exclude the specific trials where participants did not actively move the cursor. However, we believe that our exclusion criteria, which include removing subjects who missed more than 40% of catch trials, removing subjects whose control thresholds were more than three standard deviations higher than the mean threshold, and removing subjects with a non-positive d’, all could serve to exclude participants who exhibited a tendency to not move the cursor during trials.

Please see our response to comment #2d below for the revised portion of the manuscript that discusses how future studies will benefit from collecting

participants’ cursor movement data to better assess the influence of trials where subjects did not move the cursor on the accuracy of self-agency measurements.

d. Did some participants move the cursor more than others, or did the amount of cursor movement change over the course of the test?

We thank the reviewer for this valuable comment. As our response to comment #2c states, we did not collect participants’ cursor movements in the current experiments. Thus, we revised the discussion section to discuss this limitation and to encourage future studies to collect cursor movements. In addition, code that records cursor movement was made available in the online repository of the manuscript.

The following changes were made in the manuscript to address this comment:

# *The present study has ~~at least two~~ a few limitations … Second, the lack of cursor*

*position recording in the current study limited our ability to assess the influence of participants’ movement characteristics (e.g., velocity, shape of trajectories) on their measurements of self-agency. Therefore, experiment code that records mouse movements is provided in the online repository. This will allow future studies to explore the questions above.*

3. Line 46. More details about the Libet clock paradigm would be helpful. How it works, what the patient is asked to do, etc.

We agree that including more details about the Libet clock paradigm could help readers better understand the flaws of existing self-agency measures. We have modified the introduction accordingly:

# *For example, the Libet clock paradigm~~, which~~ is widely used to assess movement-based*

*self-agency in experimental settings [12]. In this task, a dot loops around a clock face with a uniform speed. While tracking the movement of the dot, participants can press a button to stop the dot with a variable delay. Depending on the instruction, they then report the location of the dot at the timing of movement intention initiation or movement*

*execution. The Libet task has been criticized for confounding perceived timing of voluntary movement initiation and execution with timing for memory processing [13], and for its general susceptibility to memory-based biases [14-16].*

4. Line 57-58. I’m not sure what this means. For readers who have no background knowledge of FMD, like me, it would be helpful to have more explanation. What effect does this mismatch have on the patient’s ability to function? How does it affect their daily lives?

We agree that providing more information on FMDs could give readers a clearer picture of the disease and a better understanding of how the loss of self-agency could impact people’s lives. To address this comment, the following changes were made:

# *Among the disorders mentioned above, we focus on FMDs here, as their diagnosis is* particularly challenging. FMDs are movement disorders characterized by body movements or postures that patients cannot control, and that have no known neurological [basis [18]](https://paperpile.com/c/sb2IIY/hp72). The most prominent feature of FMDs is ~~the~~ patients’ lack of a sense of

*agency over body movements or postures [2]. When this happens, patients feel that they do not own the behaviors their bodies carry out. In other words, their bodies exert random movements, such as tremor and twitches, that do not feel self-initiated and cannot be controlled. This is often reported to be disruptive and, in some cases, can lead to a prolonged state of disability [19].*

5. The method of assessing test-retest reliability is a little unusual. Are there any plans to have participants complete the task two separate times, at least several days apart, and assess how consistent the results are within participants, across days?

We thank the reviewer for this comment. In both experiment 1 and experiment 2, each participant only performed the task once. Thus, we only reported the stability of our measurements within the session. However, we agree that it is important to validate the task’s test-retest reliability both within-session and across-days. Hence, we revised the discussion to mention the lack of an across-days stability measure. Furthermore, in a planned future study that will compare healthy and patient populations, participants will

be encouraged to perform the task up to three times with at least one week apart between each session. We hope that this future study will allow us to better address the test-retest reliability of the current task.

In addition, we have added split-half reliability analyses on participants’ control levels on the 8th to 13th reversal trials to further support our within-session reliability analysis. In this test, we calculated the mean correlation coefficient of all split-half combinations of the six reversal trials and the reliability score. The 8th reversal trial was selected since this is usually the point when the staircases reached a stable measurement in pilot experiments. Results showed that strong reliability was observed in both experiments. To verify the robustness of this result, we extended this test to 7th to 13th, and 6th to 13th reversal trials.

The following revisions were made to address this comment:

In the result section:

# *Test-retest reliability of control level threshold estimation was assessed by evaluating the* correlation between the average of each participant’s control levels on the 8th to 10th reversal trials and the average of the subject’s control levels on the 11th to 13th reversal trials. The 8th to 13th reversal trials were selected because, based on pilot data, staircases typically reached stable values starting at the 8th reversal trial. We aimed to verify the within-session stability of the staircasing procedure by performing the correlational test above. Spearman rank correlation tests were used to minimize the influence of extreme values. Positive correlations were observed in both experiments [Experiment 1: r(51) = 0.90, p < 0.001, Fig 3A; Experiment 2: r(52) = 0.83, p < 0.001, Fig 3B].

*In addition, we calculated the split half reliability based on participants’ control levels on the 8th to 13th reversal trials. High reliability was observed in both experiments (Experiment 1: r = 0.99, reliability = 0.997; Experiment 2: r = 0.97, reliability = 0.99)[38]. Similar results were obtained if the test was performed on control levels on the*

*7th to 13th or 6th to 13th reversal trials (Experiment 1, reversals 7-13: r = 0.995, reliability = 0.998; Experiment 1, reversals 6-13: r = 0.99, reliability = 0.997;*

*Experiment 2, reversals 7-13: r = 0.97, reliability = 0.99; Experiment 2, reversals 6-13: r*

*= 0.97, reliability = 0.99).*

In the discussion section:

# *The present study has ~~at least two~~ a few limitations… Third, since data was collected*

*from each participant only once, we were not able to verify the task’s test-retest reliability within participants and across days. Therefore, future studies should further assess test-retest reliability by collecting data across sessions separated by multiple days.*

## Reviewer #2

1. The paper could give a better sense of the observed movement data – what were subjects' movement velocities? What did their trajectories look like? Did they explore the whole workspace? Was there a relationship between specific kinematic properties (e.g. average velocity) and agency thresholds?

We thank the reviewer for raising these interesting and valuable questions. Please see our response to reviewer1’s comment #2c & #2d.

2. Figure 1A is not particularly illuminating – I would suggest showing several frames of the dots moving, perhaps with example movements of the subject below it, to really illustrate the task dynamics. Otherwise it's hard to get a sense of the phenomenological experience of the subjects, which is key in studies of high-level cognitive inferences like agency.

We thank the reviewer for this comment. To address this issue, we have added a short supplementary video that shows several example trials with mouse movements

side-by-side with the dot motion stimuli.

This supplementary video was referenced in the modified Fig 1 caption:

***Fig 1. Task procedure and calculation of dot trajectories.*** *A) Task procedure. At the beginning of each trial, a fixation cross was presented for 0.5 s, followed by the dot*

# *stimuli. When the dot stimuli were on the screen, participants could influence the moving* trajectory of one of the dots with cursor movements. Then, after a blank screen (0.5 s), participants judged which of the dots they were better able to control (A or B), and how confident they were in this judgment on a scale from one to four. Font sizes are enlarged relative to their actual size during the experiment for clarity. See Video S1 for an illustration of example trials with mouse movements side-by-side with the dot motion stimuli. B) Calculation of dot trajectories.

3. For the test-retest reliability correlations, why were the particular trial bins chosen (8-10/11-13)? These should be justified in the text somewhere.

We agree that the justification for our test-retest reliability analysis should be provided. We initially chose the 8th to 13th reversal trials because the staircasing procedure usually starts to stabilize after the 8th reversal, based on pilot data. We divided the six reversals into two halves from the middle and computed the correlation to show that our staircases reached and maintained stable measurements at the end of the session.

However, we recognize that this analysis of reliability could be insufficient. Hence, we added a split-half reliability analysis, which further supports the high observed

within-session test-retest reliability (see response to reviewer 1’s comment #5).

4. Misspelled reference to "Dannett" (ref 14) I believe should be "Dennett."

We thank the reviewer for this comment and the mistake has been corrected:

# *~~Dannett~~ Dennett DC, Kinsbourne M. Time and the observer: The where and when of*

*consciousness in the brain. Behav Brain Sci. 1992;15(2):183-247. doi: 10.1017/S0140525X00068229.*

5. It could be nice in the correlation figurers (i.e. fig 3) to have rho values depicted on the figure panel.

We agree and have revised fig 3 as shown below:

**References**

1. Goodman JK, Cryder CE., Cheema A. Data collection in a flat world: The strengths and weaknesses of Mechanical Turk samples. J Behav Decis Mak. 2012;26(3):213-224. doi: 10.1002/bdm.1753.
